# Toward a Clearer Process for Value Sensitive Artificial Intelligence

**DOI:** 10.1007/s11948-026-00583-2

**Published:** 2026-02-14

**Authors:** Christina Cociancig, Hendrik Heuer

**Affiliations:** 1 University of Bremen, Am Fallturm 1, 28359 Bremen, Germany; 2 Center for Advanced Internet Studies, Konrad-Zuse-Straße 2a, 44801 Bochum, Germany; 3 University of Wuppertal, Gaußstraße 20, 42119 Wuppertal, Germany

**Keywords:** Value sensitive design, AI ethics, Value sensitive artificial intelligence, VSAI

## Abstract

Design approaches such as Value Sensitive Design (VSD) facilitate the alignment of technology design with societal and ethical values. VSD serves as a proactive and iterative framework for integrating ethical considerations into design, encompassing three interrelated types of investigations: conceptual investigations, which analyze relevant values and stakeholders; empirical investigations, which gather data about stakeholders’ experiences and value prioritization; and technical investigations, which focus on embedding these values into the functionality of technologies. This paper critically examines the application of VSD for artificial intelligence (AI) through a systematic literature review. By addressing VSD’s theoretical pillars and AI’s ambiguous boundaries, the study maps these complexities to the descriptions of the practical implementation in value sensitive AI (VSAI) literature. Our findings contribute to a comprehensive framework identifying practical and theoretical gaps, such as the operationalization of VSD’s tripartite methodology in AI contexts and value prioritization. We contribute a synthesis of recommendations from the literature as well as our own to fill these gaps and offer actionable strategies for future VSAI applications. Together, these insights clarify the current methodological landscape, and provide researchers with practical guidance in building value sensitive AI.

## Introduction

The evolution of artificial intelligence (AI) has expanded its influence across nearly all aspects of life. As AI becomes more pervasive, the urgency of designing systems that represent and ideally advocate for societal and ethical values has never been greater. This urgency stems from both the transformative potential and the risks associated with AI use. A design methodology that emphasizes a proactive, value-driven approach is Value Sensitive Design (VSD) (Friedman & Hendry, 2019). It is based on the tripartite process of investigations with different focuses: Conceptual investigations identify the values implicated by a technology, empirical investigations gather data on affected stakeholders’ perspectives, and technical investigations examine how either existing technologies implement values (retrospective) or new technologies should implement them (proactive).

Building on this foundation, several frameworks have emerged that adapt and extend the methodology to address the unique challenges of designing AI systems. For example, by prescribing methods and processes in more detailed frameworks (Zicari et al., 2021), by focusing on the design of certain aspects of an AI system like the algorithm (Zhu et al., 2018), or by modifying the design phases to adapt to existing ethical frameworks (Umbrello & Van De Poel, 2021).

While these adaptations benefit from VSD’s flexibility in implementation, they often deliberately avoid core methodological challenges. VSD has faced significant critique from the research community (Manders-Huits, 2011; Borning & Muller, 2012; Davis & Nathan, 2015; Reijers & Gordijn, 2019; Gerdes & Frandsen, 2023; Sadek et al., 2023) on challenges with the human values that VSD promotes, the lack of an explicit normative background, and gaps in method application. These critiques create uncertainty about how to apply the methodology in practice, leaving researchers to interpret VSD’s principles and intentions in often subjective manners.

Instead of modifying VSD, this study examines the methodology in its original form. By identifying and addressing its inherent challenges, we seek to provide a clearer picture of how the theory of VSD correlates with its practice, and how challenges can be addressed going forward. This approach ensures that future research in addressing ethical considerations in AI with VSD can be better informed while preserving the core pillars of the methodology. To this end, we explore the following research questions:

- **RQ1**: What is the current state of applying VSD to AI as reported in research studies?
- **RQ2**: Which approaches have been proposed in the literature to address challenges in applying VSD to AI?

A systematic literature review (SLR) of publications applying VSD to AI was conducted to address these research questions. The primary aim of this paper is to constructively observe challenges in the methodology itself as well as its application, but in the spirit of contributing to VSD’s overall impact on value sensitive AI (VSAI). It builds on the findings of Winkler and Spiekermann (2021) in a study of 17 publications that apply the VSD methodology to non-AI technology. Instead of examining publications that deploy the *full* tripartite methodology of VSD for *any* technology only, this paper focuses on AI systems, and considers partial implementation of the tripartite methodology (e.g., studies that consist of only a conceptual investigation) as well. Rather than adopting an a priori definition of the term “AI”, we examine how AI is conceptualized in VSAI. For inclusion into our corpus, we considered studies examining AI applications (e.g., chatbots, drones, robotics) and techniques (e.g., machine learning, NLP, image recognition, augmented/virtual reality) or synonymous concepts (e.g., autonomous systems, algorithm). We do not aim to promote VSD as the definitive solution for addressing ethical issues in AI systems, rather, we seek to outline a clearer process for this methodology. Building on our findings, we have developed a framework that takes a holistic approach to VSD challenges, both general and AI-specific, providing VSAI researchers and practitioners with a structured guide to anticipate and address practical limitations.

## Background

VSD is a design methodology aimed at integrating human values into the development of technology. Its origin lies in communities in computer ethics, social informatics, computer-supported cooperative work, and participatory design (Friedman & Hendry, 2019). In the 1990s, VSD emerged to answer the need for a universal design approach that highlights the sociotechnical relationship between technology and the human, more specifically the user or “stakeholder” of a technology. As an evolving methodology, it is assembled on the pillars of a proactive orientation toward influencing design, and the resistance of prescribing ethical theories while providing a heuristic of human values impacted by design.

### Pillar 1: Proactively Influencing Design

A central pillar of VSD is its proactive orientation towards influencing design. This is achieved by integrating ethical considerations from the beginning and *iterating* through design phases, which allows for continuous refinement and adaptation. The investigations (i.e., conceptual, empirical, and technical) can be integrated and repeated at any point in the design cycle. VSD draws on a multidisciplinary collection of methods to conduct these investigations (Friedman et al., 2017), including methods from anthropology, design, psychology, philosophy, and software engineering. With this breadth of methodological approaches as an explicit goal, VSD is flexible and broadly applicable, but ultimately, as a methodology, loses some of its robustness. Friedman and Hendry (2019) refer to this as a “hard problem” of VSD, which resulted from their strategic decision to *not specialize* in a particular value, technology, population, or context. Therefore, any theory backing the use of the methodology or any method applied within VSD investigations, needs to be carefully considered and evaluated by those who work with it.

### Pillar 2: Providing a Heuristic

VSD does not comply with a specific normative framework or ethical theory, because moral discourse is “rife with disagreement” anyway (Friedman & Hendry, 2019). VSD’s aspiration is to provide a *heuristic*, including a list of *human values* that are impacted by technology design. The conceptualization of a human value as what is “important to people in their lives, with a focus on ethics and morality” (Friedman & Hendry, 2019) is intentionally broad, though still centering the moral source. The list of human values was developed by Friedman and Kahn (2003) and consists of: human welfare, ownership and property, privacy, freedom from bias, universal usability, trust, autonomy, informed consent, accountability, courtesy (added later (Friedman et al., 2006)), identity, calmness, and environmental sustainability. Friedman and Hendry acknowledged the incompleteness and the values’ roots in deontology and consequentialism (Friedman & Hendry, 2019), however, this list is intended as a starting point to determine implicated values. VSD’s position on the universality of these values was initially (in the early 2000s) vaguely permissive (Friedman & Kahn, 2003), later, certain that *some* values are universal (Friedman et al., 2006), while then claiming a softer position by stating: “On the question of universal values […] value sensitive design does not articulate a definitive answer” (Friedman & Hendry, 2019).

### The Gap Between Theory and Practice

Despite efforts to maintain conceptual robustness of the two pillars, the application of VSD in practice in many cases is often considered challenging. The gap between theory and practice does not only pertain to principles of the methodology which are not carried into application, but also theory choices that are critiqued rather than accepted in practice.

#### Challenges with Pillar 1

While the tripartite nature of VSD in combination with iteration is the central concept to VSD’s proactive orientation towards influencing design, both have been subject to critique and challenges in application. Reijers and Gordijn (2019) claim these investigations narrow the understanding of the potential impact of technologies, because they omit broader societal and systemic implications. Moreover, by integrating empirical methods with conceptual research, VSD risks committing the naturalistic fallacy (“is” vs “ought”) by relying on empirical knowledge to inform normative decisions (Manders-Huits, 2011). In practice, this combination and sequence of conceptual investigation followed by empirical investigation is found to be the most prevalent in VSD research (Winkler & Spiekermann, 2021). Undermining the tripartite nature of the methodology, technical investigations are frequently neglected in VSD research (Winkler & Spiekermann, 2021; Gerdes & Frandsen, 2023). Iterations over investigations are neglected as well: Winkler and Spiekermann (2021) reviewed 17 VSD projects and revealed that only four explicitly reported iterative processes over any or all investigations. Beyond the structural limitations of the tripartite iterative structure, further issues arise at the level of the specific methods employed within each type of investigation. In conceptual investigations, the crucial step of stakeholder identification is not clearly guided by VSD (Manders-Huits, 2011). If conducted, technical investigations rarely rely on the input of stakeholders, but on the expertise of researchers (Winkler & Spiekermann, 2021) who interpret and “translate” the results of conceptual and/or empirical investigations without standardized criteria.

#### Challenges with Pillar 2

The ethical non-commitment of VSD (Manders-Huits, 2011, Davis & Nathan, 2015, Gerdes & Frandsen, 2023) has also been addressed by Friedman and Hendry (2019) who consider it as adaptability of the methodology, and thus a motor to advance it. This motor has been utilized, e.g., by van Wynsberghe (2013) whose research in value sensitive design of robots for healthcare is rooted in care ethics. However, this aspect of adaptability is simultaneously cause for challenges: It leaves room for subjective interpretation of scope and methods of VSD by researchers. Additionally, it influences how human values are identified and applied within VSD. While VSD refrains from providing a definitive answer to the universality of human values, the heuristic list has also been critiqued to be incomplete and arbitrarily defined, limiting its applicability across diverse contexts (Reijers & Gordijn, 2019; Sadek et al., 2023). Compounding this issue is the lack of value embodiment within VSD: Values are treated as non-normative, offering no clear prescriptions for how they should guide decision-making in the design process (Sadek et al., 2023). Furthermore, the approach to integrating values in VSD frequently relies on top-down elicitation methods, where pre-existing lists of values are applied rather than being elicited from stakeholders (i.e., bottom-up), diminishing the inclusivity and relevance of the process of value identification itself (Sadek et al., 2023). Contrary to this finding for VSD, in VSAI research, a mix of top-down and bottom-up value elicitation can be identified (Sadek et al., 2023). The applicability of a value heuristic developed for the technology of the 1990s reaches inherent limits with AI. How can the dynamic nature of human values and continuous learning of some AI systems align (Gerdes & Frandsen, 2023)? To address some of these issues, Borning and Muller (2012) have proposed to contextualize existing and future value lists such as the human value list of VSD.

## Method

We conducted a systematic literature review (SLR) following the guidelines of Kitchenham and Charters (2007) and Snyder (2019), and combining qualitative content analysis guided by Mayring’s approach (Mayring, 2015; Mayring & Fenzl, 2019) with quantitative content analysis. Our goal was to identify both methodological practices and challenges that recur across VSAI studies.

We searched SCOPUS, one of the largest peer-reviewed databases with broad disciplinary coverage, and exported the results on May 10, 2024, and updated on January 10, 2025. We used the query “value sensitive design”, restricted to searches in abstracts, to ensure that the identified studies explicitly engage with our study’s focus. Unlike titles or keywords, which can include broader terms, abstracts offer a more detailed account of the research scope and relevance. This search resulted in 474 studies published between 2000 and 2024. We screened abstracts and, where inconclusive (*n* = 81), conducted full-text screenings. After screening, we applied strict exclusion criteria that ensured comparability between the studies, to retain our focus on VSAI studies that *apply investigations* to an *AI* system *without altering* VSD’s foundational principles. Table 1 summarizes the inclusion and exclusion criteria. The selection process is documented using the Preferred Reporting Items for Systematic reviews and Meta-Analyses (hereafter: PRISMA) (Page et al., 2021). PRISMA, developed for the healthcare context (Rogers & Seaborn, 2023), allows a high degree of transparency and thus reproducibility of SLRs. Our final corpus consisted of 37 VSAI studies. Details on the included studies are provided in Appendix A.1, the PRISMA flow chart in Appendix B.1.

**Table 1:** Inclusion and exclusion criteria for the SLR. “R1”-“R6” stand for *R*easons for exclusion within the screening process

Inclusion Criteria	Exclusion Criteria
The study reports on an empirical and/or technical and/or conceptual investigation based on the VSD methodology. The examined technology is considered within the context of AI.	Duplicate records Citations of whole proceedings Non-English reports R1: No (unpaid) access to abstract and/or full paper R2: No VSD investigations were applied R3: Study extends or integrates (parts of) VSD in its own methodology R4: Study does not apply VSD to a technology R5: Study uses “value sensitive design” descriptively R6: Study appeared in search results but does not fulfill formal criteria Study is outside of the AI context

To analyze the material, we conducted a qualitative content analysis (Mayring, 2015; Mayring & Fenzl, 2019) in MAXQDA, supplemented by quantitative data. In Mayring’s approach to qualitative content analysis (Mayring, 2015; Mayring & Fenzl, 2019), codes reflect concepts or patterns identified in the data, which can be refined through clustering. We began with an open, inductive strategy and refined our coding guideline iteratively. The first author conducted the initial coding, the second author reviewed the coding, and within a discussion an agreement was reached. After approximately ten studies, no new codes emerged, but we consolidated and clustered categories. We used MAXQDA’s quantitative tools to examine code frequencies and code crosstabulations. AI-assisted functions were not used for this study.

## Results

For reporting results to RQ1 on the current state of applying VSD to AI, we draw on VSD’s pillars from Section “The Gap Between Theory and Practice”, and present how they are operationalized in VSAI research. For reporting results for RQ2 on approaches to challenges, we draw on the challenges for VSD outlined in Section “The Gap Between Theory and Practice” and any additional challenges specific to VSAI.

### RQ1: What is the Current State of Applying VSD to AI as Reported in Research Studies?

#### Systematically Integrating Values into Design

The use of VSD for AI design is motivated by its ability to “integrate values of ethical importance” (Bozdag & Van De Poel, 2013) by providing a “bridge between analyzing ethical issues and the technical engineering design process” (Cummings, 2006). VSD is referred to as a “principled” (Capasso & Umbrello, 2022; Debrabander & Mertes, 2022; Ransan-Cooper et al., 2022) and/or “systematic” approach (Cummings, 2006; Chen & Zhu, 2019; Badillo-Urquiola et al., 2020; Capasso & Umbrello, 2022; Chen et al., 2022; Debrabander & Mertes, 2022; Ransan-Cooper et al., 2022; del Valle JI & Lara, 2023) throughout VSAI studies, though the specifics of its “principles” or “system” remain undeclared. Its retrospective or prospective application is considered a strength by VSAI research (Cawthorne & Robbins-van Wynsberghe, 2019; Thornton et al., 2019; Capasso & Umbrello, 2022; Hinton, 2023; Umbrello et al., 2024). The methodology is accessible for non-experts (Cummings, 2006; Capasso & Umbrello, 2022), thus additionally fostering cross-disciplinary collaboration (Ransan-Cooper et al., 2022; Umbrello et al., 2024) with AI researchers.

#### Coverage of Tripartite Methodology and Their Methods

A majority of VSAI research consists of or includes a conceptual investigation (*n* = 34/37) and most conduct the full tripartite methodology of conceptual, empirical and technical investigations (*n* = 21/37). Only one publication (*n* = 1/37) reports on an iteration of one or more design phases. This section presents a detailed analysis of our findings for each of the three investigation types.

*Conceptual Investigations*: Though the order of investigations is not prescribed, conceptual work in VSAI often precedes empirical and technical investigations, because it lays the groundwork on which values and groups of stakeholders are implicated by a design. Identifying stakeholders is integral to conceptual investigations and in the examined VSAI studies mostly done via “stakeholder analysis” (Chen & Zhu, 2019; Thornton et al., 2019; Hinton, 2023). This crucial process is reported as a stand-alone method to “identify individuals and groups” that are affected by a technology or its use (Tuomela et al., 2019). The specifics of how stakeholder analyses are conducted are rarely made explicit. Most conceptual investigations that conduct value identification as conceptual investigations consist of literature-based work (Hayes et al., 2020; Mecacci & Santoni de Sio, 2020; Lüthi et al., 2021; Robertson et al., 2021; Chen et al., 2022; Debrabander & Mertes, 2022; Smits et al., 2022b; Kerr et al., 2023; Riebe et al., 2023; Sonntag et al., 2023; van der Hoek S et al., 2023). To then validate that the identified values are relevant to the identified stakeholders, VSAI applies methods such as focus groups (Riebe et al., 2023), workshops (Sonntag et al., 2023), interviews (Smits et al., 2022a, b), or participatory evaluation (Ransan-Cooper et al., 2022). However, conceptual investigations draw on interdisciplinary methods, for VSAI this includes computational methods (Chen et al., 2023), economic tools (Boshuijzen-van Burken et al., 2023) or policy making (Boshuijzen-van Burken et al., 2024). Addressing and resolving value tensions, such as safety versus privacy, is an explicit goal of conceptual investigations, however, also rarely executed by applying an explicit method. One exception to this tendency can be found in Boshuijzen-van Burken et al. (2024) who conduct a participatory value evaluation which this study uses to rank value preferences by clustering and evaluating the importance of values on scales. In other studies, values are organized using tools such as hierarchical value maps (Tsunetomo et al., 2022) or envisioning cards (Iversen et al., 2020), which provide some structure to abstract lists of abstract values.

*Empirical Investigations*: Methods applied in empirical investigations include a combination of qualitative research methods such as (semi-structured) interviews, focus groups and collaborative/participatory methods (Chen & Zhu, 2019; Tsunetomo et al., 2022; Riebe et al., 2023; Sonntag et al., 2023); as well as quantitative approaches such as surveys and statistical analysis (Chen et al., 2023). Novel or rather uncommon methods are also applied, e.g., sensory ethnography (Tuomela et al., 2019) or community-based co-design (Elsayed-Ali et al., 2020). Empirical investigations also integrate phases of testing, evaluation, and refinement (Chen & Zhu, 2019; Smits et al., 2022b) of systems, algorithms and their prototypes.

*Technical Investigations*: Technical investigations in VSAI literature often lack transparency and AI-specificity, with researchers using vague terms like “technical analyses” (Bozdag & Van De Poel, 2013) or “prototype developments” (Chen & Zhu, 2019) without detailing their methods. This mirrors the lack of clarity surrounding one of the most common approaches to technical investigations: translation of values into design requirements. Most technical investigations involve this “translation” task (Bozdag & Van De Poel, 2013; Robbins & Henschke, 2017; Mecacci & Santoni de Sio, 2020; Boshuijzen-van Burken et al., 2023; Kerr et al., 2023). Predefined methods for a structured approach to this task are rare, most descriptions of implementations of this task remain at the level of “we translate this value into norms, which can be used as design requirements” but “usually more than one translation is possible” (Bozdag & Van De Poel, 2013), highlighting the subjectivity involved in this task. More structured approaches to technical investigations implement value hierarchies (Mecacci & Santoni de Sio, 2020; Umbrello, 2020; Capasso & Umbrello, 2022), prototyping with paper or digital mockups (Tsunetomo et al., 2022), and comparative analyses with similar systems (Hinton, 2023). Keeling et al. (2019) describe the outcome of the technical investigations as: “every design choice is connected back to values from the conceptualization phase”. For those technical investigations that include iteration, prototypes are developed, tested, and refined based on empirical feedback from users (Chen & Zhu, 2019; Thornton et al., 2019) or designers (Tsunetomo et al., 2022) in the form of focus groups (van der Hoek S et al., 2023) or user surveys (Sonntag et al., 2023). Some studies collect feedback on systems or prototypes thereof from users but refrain from redesigning at this point and plan to do so in the future.

#### Technology Agnosticism Put to the Test

VSD intently does not specialize in a specific technology. Therefore, it is interesting to examine methodological as well as linguistic differences in descriptions of VSAI research to investigate the scope and adaptability of the methodology. “AI” in VSAI research is vaguely described in many cases (e.g., by just using “AI” and not further specifying, “AI-driven technologies”, “intelligent” or “smart” systems, etc.). In an attempt to conceptualize and contextualize AI, we inductively categorized the system types as they appear in the corpus: The most prevalent form of VSAI studies examine “machine learning” (Chen & Zhu, 2019; Badillo-Urquiola et al., 2020; Debrabander & Mertes, 2022; Hinton, 2023; Kerr et al., 2023; Riebe et al., 2023; del Valle JI & Lara, 2023), associated techniques like data filtering (Badillo-Urquiola et al., 2020), and systems like recommender systems (del Valle JI & Lara, 2023). Not all recommender systems are explicitly based on machine learning, some are referred to as recommendation algorithms (Chen & Zhu, 2019) or automated recommendation (Cummings, 2006) not closer specifying the underlying systems. “Autonomous systems”, including autonomous vehicles (Thornton et al., 2019; Mecacci & Santoni de Sio, 2020; Chen et al., 2023), drones (Iversen et al., 2020; Boshuijzen-van Burken et al., 2024), and missiles (Cummings, 2006) are also a prevalent description for the system type in VSAI research. Human- or consumer-centered AI systems like chatbots (Hinton, 2023) and several digital (voice) assistants (Elsayed-Ali et al., 2020; Hinton, 2023) signal a growing emphasis of ethical considerations for AI systems with direct human interaction. VSD is also applied to specialized and emerging technologies like augmented (Friedman & Kahn, 2000; Deng & Christodoulidou, 2015; van der Hoek S et al., 2023) and virtual reality (Elsayed-Ali et al., 2020; Smits et al., 2022a; Sonntag et al., 2023) or quantum technologies (incl. quantum machine learning) (Umbrello et al., 2024). Only one study mentions generative AI (del Valle JI & Lara, 2023).

Despite the differentiation between AI techniques and applications, our analysis reveals that these distinctions are not reflected in the methodological application of VSD. Across the literature, we found that VSAI studies do not adapt their research design to the type of AI system under investigation, even though there could be potential for modifications for specific AI system types or techniques, e.g., as in Value-Sensitive Algorithm Design (Zhu et al., 2018).

#### Representation of Norms and Ethical Theories

VSD is not based on a specific normative or ethical theory - a shortcoming diminishing the methodology’s robustness for some critics as explained above. However, in VSAI research studies, some tendencies can be extracted. Two publications in our corpus derive their values from explicit ethical theories: Debrabander and Mertes (2022) base their investigated values on the unified theory of rational autonomy by Pugh (2020), and Mecacci and Santoni de Sio (2020) base theirs on the theory of meaningful human control by Santoni De Sio and Van Den Hoven (2018). Nonetheless, aligning with VSD’s roots in consequentialism and deontology (Friedman & Hendry, 2019), most VSAI research outlines ideas and motivations that represent those schools of thought.

The philosophy of consequentialism surfaces in VSAI research, when the argument focuses (with a positive or negative connotation) on the outcome of the use of AI. In most studies, the authors establish their motivation for proactively addressing human values in design with consequentialist considerations of the duality of benefits versus risks and harms of AI. Hayes et al. (2020) analyze this duality of AI as a great potential for benefits “but also a significant capacity to cause harm”. As benefits, Hayes et al. (2020) recognize the “power to produce insights” and Hinton (2023) sees a “potential to change various aspects of citizens’ daily lives and of society as a whole”. Others highlight specific benefits like an increase of efficiency (Riebe et al., 2023), performance in categorization tasks (Robbins & Henschke, 2017) and AI’s general ability to fulfill a task “as well as the person” (van der Hoek S et al., 2023). In contrast to AI’s benefits, ethical concerns and risks are mentioned almost in the same breath. Critical metaphors, like “algogracy” to describe the growing influence of algorithmic systems on society (Hayes et al., 2020), or “stealing data” and “ruling the world” (del Valle JI & Lara, 2023), underscore the societal concerns about AI. Harms that are feared are diverse: exposure to toxic content online (Badillo-Urquiola et al., 2020), polarization (Bozdag & Van De Poel, 2013), bias (Smits et al., 2022b), threats to human autonomy (Debrabander & Mertes, 2022; del Valle JI & Lara, 2023), and skill degradation (Cummings, 2006), to name a few examples.

The philosophy of deontology emphasizes adherence to moral rules or duties, judging the morality of actions based on their intrinsic nature rather than their consequences. These considerations arise when authors focus on giving a voice to vulnerable groups of people: children (Badillo-Urquiola et al., 2020; Elsayed-Ali et al., 2020), patients (Capasso & Umbrello, 2022; Debrabander & Mertes, 2022), or unorganized affected stakeholders (Boshuijzen-van Burken et al., 2023). Implicitly this expresses the duty of respect for others, empowering users and maintaining (human) autonomy (Umbrello, 2020; Capasso & Umbrello, 2022; del Valle JI & Lara, 2023). Similar to consequentialist considerations, deontological argumentation recognizes the duality of AI. AI is described as “transformative” (Cawthorne & Robbins-van Wynsberghe, 2019; Hayes et al., 2020; Vernim et al., 2022) and enhancing human decision-making (Chen et al., 2022; Hinton, 2023). Particularly in fields like healthcare (Felzmann, 2021; Capasso & Umbrello, 2022; Debrabander & Mertes, 2022; Smits et al., 2022a, b; Kerr et al., 2023), personalized services (Bozdag & Van De Poel, 2013; Deng & Christodoulidou, 2015; Chen et al., 2023; del Valle JI & Lara, 2023) and justice (Hayes et al., 2020), AI’s beneficence is highlighted. On the other side, descriptions such as “black box” (Bozdag & Van De Poel, 2013; Chen & Zhu, 2019; Hayes et al., 2020; Kerr et al., 2023) and “opaque processes” (Robbins & Henschke, 2017; Cawthorne & Robbins-van Wynsberghe, 2019; Badillo-Urquiola et al., 2020; Vernim et al., 2022; del Valle JI & Lara, 2023) identify challenges of AI in transparency and accountability that directly contradict our moral duties of care, respect, and autonomy.

#### Values in VSAI Research

Values are a dynamic and evolving compass for design (Smits et al., 2022b; Chen et al., 2022) and emerge from two approaches: bottom-up, where values are derived directly from empirical observations; and top-down, where they are informed by literature or the researchers’ normative lens. Context specificity is expressed bottom-up by domain-specific needs of users, e.g., by emphasis on privacy of data in healthcare (Capasso & Umbrello, 2022) in comparison to the privacy of data of smart home devices (Umbrello, 2020). van der Hoek S et al. (2023) add: “Context does influence some of the values in terms of prioritization”. Users express their immediate needs in real-world contexts, often adding dimensions like in “*teen* autonomy” and “*teen* privacy” (Badillo-Urquiola et al., 2020) representing the affected user group, or by adding the application domain like in “*traffic* safety” (Chen et al., 2023). Some top-down values are explicitly based on aforementioned ethical theories, or standards and frameworks such as the UN Sustainable Development Goals (Capasso & Umbrello, 2022). They promote and represent consistency in values applied across contexts and domains, e.g., fairness, accountability, explainability, autonomy, and non-maleficence (Cawthorne & Robbins-van Wynsberghe, 2019; Iversen et al., 2020; Capasso & Umbrello, 2022; Hinton, 2023).

**Fig. 1:**
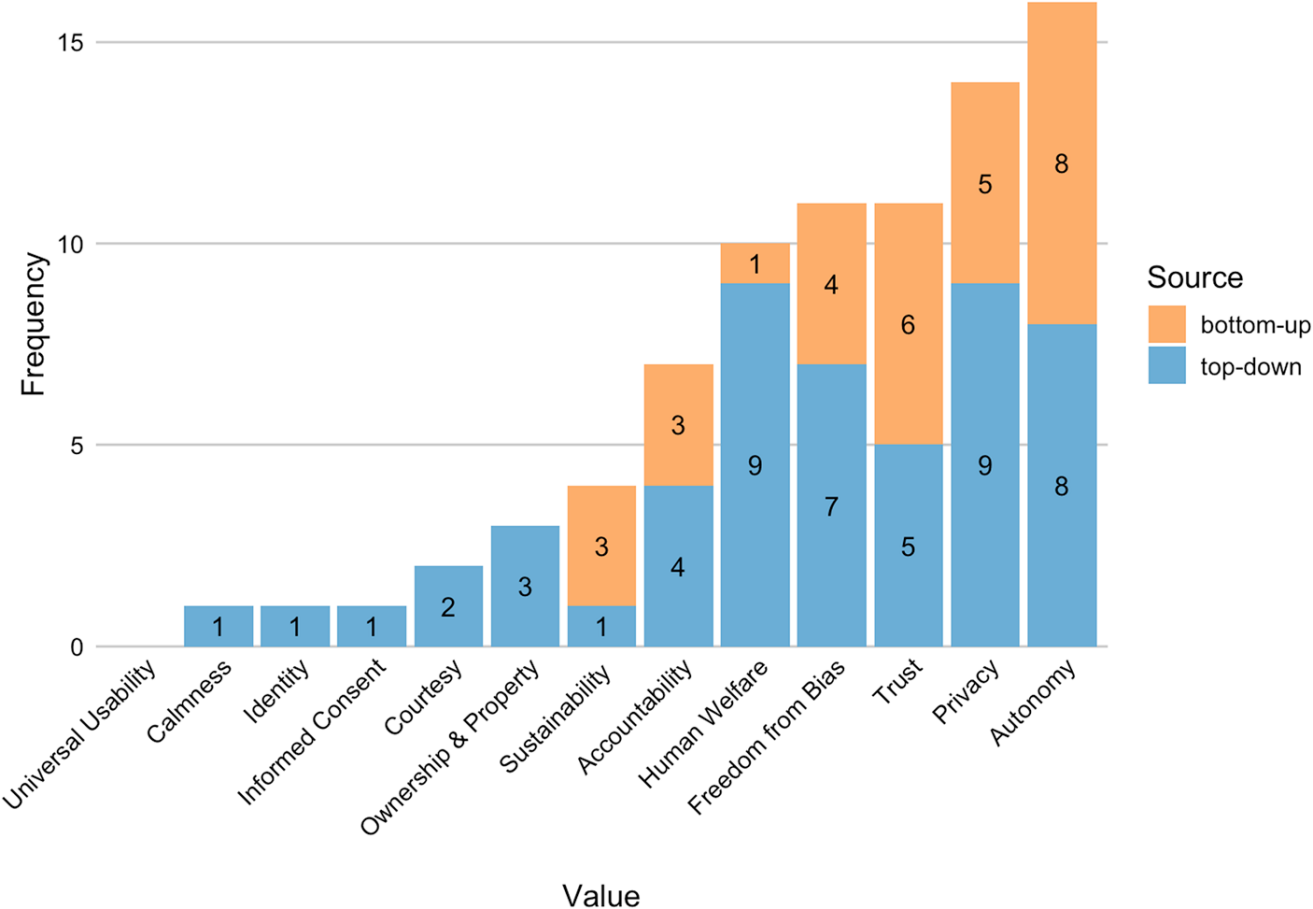
Frequency of the 13 human values of VSD in VSAI studies in ascending order. The stacked bars show the ratio of bottom-up (orange) and top-down (blue) emergence of each value

Fig. 1 visualizes the representation of human values and their sources in our corpus. VSD establishes 13 human values impacted by design: human welfare, ownership and property, privacy, freedom from bias, universal usability, trust, autonomy, informed consent, accountability, courtesy, identity, calmness, and environmental sustainability (Friedman & Hendry, 2019).

Across the 37 evaluated research studies, autonomy (*n* = 17/37) and privacy (*n* = 15/37) are the most frequently addressed values, while universal usability is not addressed at all. Most values are exceedingly addressed top-down instead of bottom-up. However, the ratio between the two source types varies across individual values. For example, while human welfare and freedom from bias are weighted toward top–down elicitation, autonomy and trust show a relatively balanced representation of top–down and bottom–up.

VSD deliberately does not specialize in any context, but an analysis of the domains in our corpus provides further insight into the prioritization of values within VSAI. The examined research studies describe the use of AI in domains with high ethical stakes, such as healthcare and public services, but also domains that require direct human interaction such as chatbots and augmented and virtual reality. Healthcare-related applications include clinical decision support systems (Debrabander & Mertes, 2022), wearable technology (Deng & Christodoulidou, 2015), and digital health tools (Kerr et al., 2023). The focus on this domain reflects the ethical complexity of healthcare technologies, where autonomy (*n* = 5/7) and privacy (*n* = 4/7) were frequently addressed. For transportation technologies, such as drones and automated vehicles (Keeling et al., 2019; Iversen et al., 2020), the value of safety (*n* = 3/4) dominates. Reflecting interest in integrating VSD into consumer-facing and commercial technologies, smart home systems (Tuomela et al., 2019), consumer platforms (Chen et al., 2023), VR (Elsayed-Ali et al., 2020; Smits et al., 2022a; Sonntag et al., 2023) and AR (Friedman & Kahn, 2000; Deng & Christodoulidou, 2015; van der Hoek S et al., 2023) are scrutinized. Security was the most discussed value in this domain (*n* = 3/9). Public services, including justice and security, demonstrate the application of VSD to domains with broad societal impact. In this domain, transparency and autonomy were equally prominent (*n* = 4/5 each). The same applies to military technology (Cummings, 2006; Boshuijzen-van Burken et al., 2023, 2024).

The variety of values represented in our corpus go beyond the heuristic of 13 human values that VSD establishes. Most values in these research studies are moral values, reflecting on what is considered right or just. Apart from the human values defined in VSD like fairness (Capasso & Umbrello, 2022; Hinton, 2023) or (respect for) human autonomy (Deng & Christodoulidou, 2015; Cawthorne & Robbins-van Wynsberghe, 2019), more values like non-maleficence (Cawthorne & Robbins-van Wynsberghe, 2019; Hinton, 2023), justice (Umbrello, 2020; Hinton, 2023), and reliability (Boshuijzen-van Burken et al., 2024) emerge. Some values implied in AI design also have procedural consequences and are not directly rooted in moral convictions. Examples are transparency (Lüthi et al., 2021; Chen et al., 2022) or accountability (Hinton, 2023; Riebe et al., 2023). Values that would be more functional than moral, like efficiency (Tuomela et al., 2019; Umbrello, 2020), reliability (Lüthi et al., 2021; Boshuijzen-van Burken et al., 2023), or adaptability and learning capability (Boshuijzen-van Burken et al., 2023) are also considered values relevant to AI design and either emerge top-down or bottom-up.

Our analysis in this section provides a detailed account of how VSD is currently applied to AI, as per RQ1. The findings demonstrate that while VSD is applied to various forms of AI, the operationalization of its core pillars remains *inconsistent*. Key gaps include the *lack of iteration* over investigations, and *underdefined processes* for stakeholder identification and translation of values into design specifications. VSD’s roots in consequentialism and deontology are commonly surfacing in VSAI, highlighted by the duality of benefits versus risks of AI. Moreover, the current VSAI research is challenged by a lack of *context* to embed values and add nuance to the analysis.

### RQ2: Which Approaches Have Been Proposed in the Literature to Address Challenges in Applying VSD to AI?

The approaches to challenges in applying VSD to AI unite two dimensions: general recommendations for advancing the methodology, and AI-specific challenges that require adaptation of VSD principles to the particular characteristics of AI systems.

General recommendations for advancing the methodology are motivated by what researchers currently struggle with in the examined VSAI studies. One recurring reported issue of applying VSD is its *limited procedural guidance*. VSD does not prescribe design phases, leading to insecurity among researchers about where to start in AI development projects (Tuomela et al., 2019; Smits et al., 2022b). Similarly, there is no systematic approach for identifying stakeholders, which additionally is a context- and time-specific step and consequently needs to also be conducted iteratively (Umbrello et al., 2024). Suggestions to support this step include the integration of other sources like public discourse and forums to diversify stakeholder perspectives (Umbrello et al., 2024). VSD also lacks a systematic (i.e. especially ongoing and direct (Robertson et al., 2021)) approach to adequately involve stakeholders in the design process (Tuomela et al., 2019; Umbrello et al., 2024), however, the suggestion here is to integrate methods from other disciplines to capture stakeholders’ values (Umbrello et al., 2024). Nonetheless, empirical work with stakeholders remains resource-consuming and has to rely on small sample sizes (Cawthorne & Robbins-van Wynsberghe, 2019, Tuomela et al., 2019, Elsayed-Ali et al., 2020, Lüthi et al., 2021, Hinton, 2023), thus also being prone to sample bias (Tuomela et al., 2019; Badillo-Urquiola et al., 2020; Robertson et al., 2021; Kerr et al., 2023). Technical investigations lack procedural guidance for the translation of conceptual and empirical findings into actionable design recommendations (Smits et al., 2022a; Umbrello et al., 2024).

Another frequently reported issue in the examined VSAI studies concerns the ambiguity and subjectivity surrounding the identification and prioritization of *values*. This is reflected in VSD’s foundational definition of human values as “what people consider important” (Friedman & Hendry, 2019) which is broad, leaving it open to subjective interpretation on what constitutes a value and how can it be operationalized (Debrabander & Mertes, 2022; Ransan-Cooper et al., 2022; Hinton, 2023). Providing a finite (though heuristic) list of human values implied by design could potentially overlook values that are context-specific (Smits et al., 2022a; Kerr et al., 2023) or even specific to geographic location and characteristics of stakeholders and technology systems (Boshuijzen-van Burken et al., 2024). Ransan-Cooper et al. (2022) highlight the absence of an “ethical yardstick” of some or any ethical theory to determine which values should be prioritized in design. Badillo-Urquiola et al. (2020) and Boshuijzen-van Burken et al. (2023) warn that the prioritization of values within design projects could transform into a framing effect of the whole investigation. Researchers should, therefore, transparently report their own values to offer a complete and transparent examination of who and what shapes VSAI investigations (Smits et al., 2022a).

AI-specific challenges require adaptation of VSD principles in value elicitation, stakeholder participation, and system design. First, traditional empirical methods fail to account for AI-specific dynamics such as personalization or data-driven system evolution (Chen et al., 2022; Hinton, 2023). These AI-specific characteristics introduce additional layers of variability and contextual dependency for value elicitation that are currently not considered. Moreover, more work is needed to assess how stakeholder’s values change over time, especially after deployment (Vernim et al., 2022), and how AI may reshape the alignment with values long-term (Robertson et al., 2021). Second, because of the AI-specific dynamics, ongoing participation of direct and indirect stakeholders, starting at the beginning of the design cycle (Vernim et al., 2022) should be highlighted. Finally, AI systems need to be designed with a stakeholder-centric mindset (Robertson et al., 2021; Hinton, 2023). For this, transparent and accountable algorithms are needed (Riebe et al., 2023; del Valle JI & Lara, 2023) that are understandable and interpretable to stakeholders, so that they can make meaningful design choices. However, Chen and Zhu (2019) concede that a “mapping between a wide range of human values and a variety of algorithmic design options is challenging” and requires expertise in the development of AI systems (e.g., to make choices in areas of pre-processing, model regularization, and post-processing), which some stakeholders lack. If the use of interpretable algorithms is not possible, opaque ones should only be used in “situations in which it is acceptable to not have an explanation or to supplement the decision of the algorithm with human oversight” (Robbins & Henschke, 2017). To operationalize the stakeholder-centric mindset, AI systems could include an element of adaptability to stakeholder feedback (Robertson et al., 2021) or modular design features that can be activated or deactivated by users (Kerr et al., 2023) if they do not agree with the represented values.

In response to RQ2, we identified challenges and approaches that emphasize the need for improved procedural guidance and a careful context-sensitive consideration of values in VSAI studies. Additionally, characteristics of AI systems, like personalization and data-driven system evolution, require adaptations of VSD principles. Approaches to implement these adaptations include the use of transparent and interpretable algorithms, as well as modular system design.

## Discussion

How researchers express their motivation to apply VSD to AI *aligns* with what the methodology promises: a principled, iterative process for integrating human values into technology design. However, our analysis shows that the practice frequently diverges from the motivation. The discussion that follows is structured around the key methodological and conceptual challenges identified in the previous section. The subheadings of Sections “Iteration replaces fragmentation”–“Establish context for values” reflect a proposed vision for how the field can evolve, in which we organize the findings into areas of concern and opportunity. We aggregate AI-specific and general challenges identified in the literature and structure them in Table 2 alongside proposed approaches and our own additions. This framework is intended to support researchers and practitioners in identifying and addressing methodological gaps and refining their application of VSAI. We apply this framework in a brief use case study of two AI systems in Section “Applying the Framework”.

**Table 2:** Summary of identified challenges in VSAI and their proposed approaches from the literature. We provide cross-references for the text passages containing additional recommendations in the sections “Iteration replaces fragmentation”–“Navigating conceptual and normative ambiguities in VSAI”

Identified Challenges in VSAI	Proposed Approaches in Literature	See:
**Methodology**		
• No prescribed order of investigations	• Prioritization of investigations (Smits et al., 2022b)	Iteration replaces fragmentation
• Little iteration over investigations	• No recommendation	Iteration replaces fragmentation
• Lack of normative basis	• Provide an “ethical yardstick” (Ransan-Cooper et al., 2022)	Navigating conceptual and normative ambiguities in VSAI
**Human Values in AI Design**		
• Missing context specificity of values	• No (specific) recommendation	Establish context for values
• Prescriptive top-down approach	• Transparency of researchers (Badillo-Urquiola et al., 2020; Boshuijzen-van Burken et al., 2023)	Establish context for values
• Random, incomplete list of values	• No recommendation	Establish context for values
**Conceptual Investigations**		
• Underdefined stakeholder identification process	• Context- and time-specificity (Umbrello et al., 2024)	Establish context for values
• Addressing and resolving value tensions	• Integrate methods from other disciplines, such as PVE (Boshuijzen-van Burken et al., 2024)	Establish context for values
**Empirical Investigations**		
• Challenging data collection	• Integrate other sources of empirical data, e.g., public discourse and forums (Umbrello et al., 2024)	—
**Technical Investigations**		
• Subjective translation of values into design requirements	• Alternative: Paper prototyping & digital mockups with designer feedback (Tsunetomo et al., 2022)	Iteration replaces fragmentation

### Iteration Replaces Fragmentation

Though almost all VSAI studies integrate all three investigation types and the tendency to conduct conceptual investigations first, researchers found the lack of a clear guidance on the order of investigations challenging (Smits et al., 2022b). Without committing the naturalistic fallacy of assuming what *is* done reflects what *should* be done, (A) the starting point of conceptual investigations seems to make the most sense, as they conduct “analytic, theoretical, or philosophically informed explorations of the central issues and constructs under investigation” (Friedman & Hendry, 2019), thus laying important groundwork for the other investigation types and especially for iterative processes. The lack of iteration was particularly striking and represents a gap that can be closed by (B) broadening the cross-disciplinary exchange and relieving some challenges with empirical and technical investigations. Empirical investigations are challenging for several reasons: Methods are resource-consuming, sample sizes are small, there are temporal constraints, only limited data is available, and they are prone to the naturalistic fallacy (Manders-Huits, 2011). Although these challenges are apparent, from the literature, it was not possible to reproduce a general rejection of empirical investigations in VSD for VSAI (Sadek et al., 2023). In VSD, technical investigations are neglected (Gerdes & Frandsen, 2023), however, this could also not be established for VSAI studies. Nonetheless, technical investigations navigate the difficulty of the underdefined and opaque “standard method” of translating values into design recommendations. This is a major blind spot for technical investigations in VSAI research that must be mitigated by (I) replacing or at least validating the outcome of translation tasks by more robust methods like comparative analyses (Hinton, 2023) with existing systems or digital mockups with designer feedback (Tsunetomo et al., 2022).

### Navigating Conceptual and Normative Ambiguities in VSAI

Two challenges emerge for VSAI: First, the concept of “AI” remains fluid and contested, which complicates scoping and comparability. In VSAI research, terms such as “AI-powered” and “intelligent” systems are used alongside “AI” as an umbrella for techniques such as machine learning and applications such as VR. While the EU AI Act definition of AI as a “machine-based system […] with varying levels of autonomy and that may exhibit adaptiveness after deployment, and […] infers […] how to generate outputs such as predictions, content, recommendations, or decisions that can influence physical or virtual environments” (2024) offers some structure, there is no consensus in the field. The term’s scope continues to evolve as it did in the past, e.g., with generative AI and quantum technologies like quantum machine learning. This flexibility of AI’s conceptualization has “helped the field to grow, blossom, and advance at an ever-accelerating pace” (Stone et al., 2016) by providing a general direction without a strict technological or contextual definition. Second, despite terminological distinctions between AI system types, VSAI studies do not adapt their application to the system type to reflect significant variation in behavior, opacity, or context. Whether the investigations examine, e.g., NLP or autonomous drones for military purposes, researchers tend to follow similar methodological processes. This generality may be a strength intended by VSD, but it also risks decoupling the method from the technical and ethical particularities of AI systems. This suggests a need for either extensions of the methodology (e.g., VSAI-specific toolkits) or more reflexive practices that link design processes to system characteristics (e.g., as in Value Sensitive Algorithm Design).

This layer of conceptual and methodological uncertainties is followed by second layer of ambiguity in VSAI: the absence of an explicit normative foundation. Although VSAI’s motivation to systematically introduce human values into the design process (C) *does* have consequentialist and deontological roots, these roots are rarely made explicit in practice. Researchers miss a normative theory (Gerdes & Frandsen, 2023) or an “ethical yardstick” (Ransan-Cooper et al., 2022). In response, some studies in VSAI (Mecacci & Santoni de Sio, 2020; Debrabander & Mertes, 2022) anchor their approach in specific theories. The only *practical* influence of an explicit underlying theory is that the heuristic value list of VSD is replaced by an (also finite, and arguably also heuristic) list of other values and how these should be prioritized. Inserting an ethical theory as a base to VSD simplifies the steps of value elicitation and value prioritization, which can undoubtedly be resource-consuming and tedious steps. Some scholars have progressed VSD by basing it in specific ethical theories, like van Wynsberghe (2013) has for ethics of robots based in care ethics. Our findings suggest that while VSAI can benefit from a grounding in explicit normative theories such as meaningful human control or care ethics, methodological pluralism remains essential. The decision to adopt a specific theory should remain with the researchers, guided by the context of the AI system and the implicated values. In this sense, VSAI should remain methodologically open yet reflexively explicit about any normative commitments made.

### Establish Context for Values

Leaving the argument for a universality of values behind us, we recognize a diverse representation of human values in VSAI research studies as well as the need to contextualize values to progress the methodology. Burema et al. (2023) found that specific values are especially prominent in specific sectors, but there are also several values that are relevant across sectors. The close connection between some sectors and specific values (e.g., hiring and discrimination) has not been established by the use of AI but existed before new technologies were used. The authors suggest to, e.g., (D) establish context-specific guidelines, which also Borning and Muller (2012) suggest. In VSAI, it seems to be especially conceptual investigations like Bozdag and Van De Poel (2013) conduct for the news sector, and Boshuijzen-van Burken et al. (2023) for the healthcare sector, that can help us gain an understanding of the values impacted in those domains. By (F) prioritizing context-sensitivity in a reasonably broad categorization, future work can oppose the critique of building a “narrow understanding of the potential impact of technologies” instead of focusing on the broader societal and systemic implications (Reijers & Gordijn, 2019). On a certain abstraction level, contextualized value lists could be a constructive approach to another heuristic. However, without meaningful technical conceptualizations or actionable recommendations for system design, contextualized lists might fail to add substantive value. Additionally, value tensions can be addressed by, e.g., participatory value evaluation (Boshuijzen-van Burken et al., 2024) which is a method borrowed from policy work. Friedman and Hendry (2019) suggest (H) two strategies for when values contradict each other: focus on shared action or pause and wait until a consensus is reached. Context-sensitivity can help us in prioritizing when these strategies cannot help. Further work is also needed on the heterogeneity of values that are impacted in AI design. It is not only moral values that are elicited in VSAI studies, and to this date it is unclear how we should treat functional values (e.g., efficiency) in comparison to moral values (e.g., accountability) in the context of VSD, and VSAI specifically.

Contrary to critique (Sadek et al., 2023), we did not identify a significant general preference of top-down or bottom-up elicitation methods, but some value-specific tendencies. For example, welfare was predominantly elicited top-down, while sustainability was predominantly elicited bottom-up. Most values are elicited top-down *and* bottom-up, suggesting agreement between researchers and users on their relevance. These findings emphasize the relevance of methodologies like VSAI that prioritize *whose values* matter in AI design. Nonetheless, with direct and indirect stakeholders (Friedman & Hendry, 2019), VSD does not yet consider researchers as stakeholders, who sometimes top-down determine which values are examined in their studies. In the interest of transparency, this stakeholder group (E) should be named and their values should be known (Borning & Muller, 2012), e.g., by using VSD value elicitation methods like value sketches (Woelfer et al., 2011) to transparently represent researcher’s values. It is also reasonable to consider more rigorous methods for stakeholder analyses, specifically, stakeholder identification (Manders-Huits, 2011) which currently is an underdefined process. This does not mean that there are no (G) structured and established methods to conduct this step: In the corpus, Critical Systems Heuristics (Boshuijzen-van Burken et al., 2023) and structured workshops (Riebe et al., 2023) were used, Friedman and Hendry (2019) also specifically list stakeholder tokens (Yoo, 2017), which is a toolkit to identify stakeholders. For stakeholder involvement, we can rely on well-established methodologies like participatory design (Spinuzzi, 2005) and co-design (Loi et al., 2019).

### Applying the Framework

To demonstrate how the proposed framework in Table 2 can guide practical research design decisions, we briefly illustrate its application by outlining the research design of two VSAI systems: an autonomous vehicle and a psychotherapy chatbot. Both apply the full tripartite methodology (prioritizing conceptual (Smits et al., 2022b) before empirical before technical investigations) in an iterative manner. Also, to address a recurring critique of VSD that stakeholders disclose their values while researchers’ own assumptions remain implicit (Badillo-Urquiola et al., 2020; Boshuijzen-van Burken et al., 2023), both research projects incorporate a reflexive step before initiating the investigations: researchers examine their own value commitments regarding the technology using established VSD tools such as envisioning cards (Iversen et al., 2020) and document the results in a research report.

#### Autonomous Vehicle

To add context-specificity and provide an “ethical yardstick” for this design of a VSAI system, similar to Mecacci and Santoni de Sio (2020), we draw on the theory of meaningful human control by Santoni De Sio and Van Den Hoven (2018). This normative theory stipulates that autonomous systems must both *track* relevant human moral reasons and *trace* system outcomes back to human agents (i.e., the “tracking” and “tracing” conditions that ensure human responsibility for autonomous systems). For laying the analytic and philosophical groundwork, we start with a conceptual investigation based on a literature review (as in Chen and Zhu (2019)) of work on the tracking and tracing conditions. In this SLR our goal is to identify top-down values associated with these conditions, as well as examine how they have been previously operationalized in autonomous driving contexts. This phase also defines the relevant stakeholder groups by using stakeholder tokens (Yoo, 2017). The empirical investigation extends the groundwork by empirically identifying values through secondary data sources such as public discourse and policy debates as suggested by Umbrello et al. (2024). This approach acknowledges that full empirical data collection can be resource-intensive, while still enabling bottom-up elicitation of values and thus validating and extending findings of the conceptual investigation. In the technical investigation, we translate the identified values into design recommendations and validate our translations through the creation of digital mockups and collecting feedback by identified stakeholders (Tsunetomo et al., 2022). These prototypes are a form of prospective technical investigation to ensure that an AI system embodies the identified top-down and bottom-up values, e.g., by visualizing driver override mechanisms (tracing) or user-interface alerts that explain algorithmic decisions (tracking). The three investigations types are iterated and the digital mockups are revised in every phase.

#### Psychotherapy Chatbot

Unlike the autonomous vehicle case, this VSAI design is not guided by a specific normative theory. In the conceptual investigation, first relevant stakeholders are identified by conducting a workshop internally within the research team (like in (Riebe et al., 2023)). Then, we conduct semi-structured value-oriented interviews (Friedman & Hendry, 2019) with users (Boshuijzen-van Burken et al., 2023) to elicit values implicated for AI systems in the healthcare and consumer-facing domain. Existing context-specific work, e.g., in the healthcare sector (Boshuijzen-van Burken et al., 2023), can support in validating the results from this step. The empirical investigation consists of focus groups (as in Chen et al. (2022) or Riebe et al. (2023)) with another stakeholder group, e.g., psychotherapists. Participants reflect on specific system features such as personalization, data storage, or the chatbot’s emotional responsiveness, which may introduce value tensions with values identified in the conceptual investigation. In the technical investigation, the findings of both preceding investigations are first implemented in a prototype and then compared to an existing psychotherapy chatbot. This comparison between two similar systems, like executed by Hinton (2023), provides suggestions for revisions of the prototype. As in the previous case, the three investigation types are conducted iteratively: insights from each phase inform successive refinements of the prototypes to ensure that the evolving system remains aligned with both top-down design intentions and bottom-up stakeholder values.

### Limitations

This study provides a broad overview of VSAI research studies rather than a detailed analysis of case-specific factors or fundamental discussions. For example, nuanced insights about particular domains or AI techniques and applications could differ, even though we were not able to analyze this aspect in our study in depth. However, the potential for a more nuanced understanding of those factors in VSAI research is also externally limited by the amount of VSAI research. For future work, we aim to expand our literature-based research by incorporating additional data sources beyond SCOPUS. More fundamental discussions, e.g., if VSD *needs* an ethical theory at its base or if its roots in consequentialism and deontology *need* to be explicit, could not be addressed here and were not consistent with our goal of showing a clearer path forward for this methodology and its application to AI.

The literature we were able to include for this study was limited due to our focus on an unaltered application of VSD to AI systems. The amount of research that is “inspired by” VSD, but does not apply this methodology with rigor, motivated the decision to keep the scope of this study narrow. Though we agree with VSD’s pioneers, that the adaptability of the methodology is an advantage to harmonize it with any technology, context, etc., in order to understand challenges with the methodology, we also need to start with the intentions with which it was built. That led us to strict exclusion criteria to ensure that the studies we include can provide us with insight into the “pure” VSD applied to AI. One such criterion was the focus on English publications. While this focus on English-language publications ensured methodological comparability within our corpus, it inevitably narrows the cultural and geographic scope of the findings. We excluded very few publications (*n* = 4) due to this criterion. For searching SCOPUS, the first author logged in via the institution she is affiliated with. Nonetheless, some publications in the search results were not available for (open) access (*n* = 16). In these cases, we conducted a search on Google Scholar, which also yielded no results that would allow us access without going through a paywall. The second exclusion criterion (R2) revealed itself as the knock-out criterion for many publications (*n* = 155), but this exclusion was necessary as it corresponded to the (tripartite) investigations and thus a core principle of VSD. The same applies to R3 which excluded modifications of VSD into new methodologies. R4 excluded VSD applications to anything else than technologies, e.g., energy projects, institutions, curricula, biorefineries, etc. Both R5 and R6 ensured that we only include studies that actually *apply* VSD and meet our formal criterion of being peer-reviewed publications. We determined AI context by querying “artificial intelligence”, “deep learning”, “machine learning”, “algorithm”, and “model” in the full text, which is a keyword list derived from the table of contents in (Goodfellow et al., 2016). We acknowledge that our exclusion criteria are selective and strict, however, with them, we ensure that our corpus provides insight into the methodology’s original principles and intentions.

## Conclusion

The aim was to assess the current state of applying VSD to AI as the research studies report, as well as to identify the approaches proposed to address any challenges associated with this application. The findings of the systematic literature review make several contributions: We provide a framework of current practices, including the coverage of the tripartite methodology and applied methods for each type of investigation; an overview of the representation of normative theories in VSAI; as well as an analysis of values implicated by AI. For future work in VSAI, we contribute by extracting approaches to address challenging aspects of the application of VSD for AI from literature, and synthesize them with our own suggestions. By examining these challenging aspects, we uncover weak spots in the methodology both general and AI-specific. We uncover them with the goal of enhancing the clarity and robustness of future VSAI studies. We argue that VSAI offers a structured approach to guiding AI development that actively reflects societal and ethical values by design.

## Data Availability

Not applicable.

## Appendix A Corpus of VSAI Studies

**Table:**

Paper	Domain	AI Category	**Investigation(s)** C = Conceptual, E = Empirical, *T* = Technical
(Badillo-Urquiola et al., 2020)	Applications	Autonomous Systems Machine Learning AI (unspecified) Image Recognition NLP	C
(Boshuijzen-van Burken et al., 2023)	Military	Autonomous Systems	C, T
(Boshuijzen-van Burken et al., 2024)	Military	Autonomous Systems	C
(Bozdag & Van De Poel, 2013)	Consumer	Recommendation Systems	C, T
(Capasso & Umbrello, 2022)	Healthcare	AI (unspecified)	E, T
(Cawthorne & Robbins-van Wynsberghe, 2019)	Healthcare	AI (unspecified)	T
(Chen et al., 2023)	Consumer	Algorithm Autonomous Systems Other (Intelligent Systems)	C, E, T
(Chen et al., 2022)	Consumer	Recommendation Systems	C, E
(Chen & Zhu, 2019)	Education	Learning Analytics Recommendation Systems Algorithm	C, E, T
(Cummings, 2006)	Military	Algorithm Recommendation Systems Autonomous Systems	C, E, T
(Debrabander & Mertes, 2022)	Healthcare	NLP Machine Learning	C, E, T
(del Valle JI & Lara, 2023)	Consumer	AI (unspecified) Recommendation Systems Machine Learning	C, E, T
(Deng & Christodoulidou, 2015)	Consumer	Other (Augmented Reality)	C
(Elsayed-Ali et al., 2020)	Consumer	Other (Virtual Reality)	C, E
(Felzmann, 2021)	Healthcare	Other (Robotics)	C
(Friedman & Kahn, 2000)	Consumer	Other (Augmented Reality)	C
(Hayes et al., 2020)	Public Services	Algorithm	C
**Paper**	**Domain**	**AI Category**	**Investigation(s)** C = Conceptual, E = Empirical, *T* = Technical
(Hinton, 2023)	Public Services	Other (Chatbot) Image Recognition Machine Learning	C, E, T
(Iversen et al., 2020)	Transportation	Other (Drone)	C
(Keeling et al., 2019)	Transportation	Algorithm	C, E, T
(Kerr et al., 2023)	Healthcare	Machine Learning	C, E, T
(Lüthi et al., 2021)	Public Services	Algorithm	C, E, T
(Mecacci & Santoni de Sio, 2020)	Transportation	Autonomous Systems Algorithm AI (unspecified)	C, T
(Ransan-Cooper et al., 2022)	Public Services	Algorithm	C
(Riebe et al., 2023)	Public Services	Algorithm	C, E
(Robbins & Henschke, 2017)	Public Services	AI (unspecified)	C, T
(Robertson et al., 2021)	Education	Algorithm	C, E
(Smits et al., 2022b)	Healthcare	Other (Virtual Reality)	E
(Smits et al., 2022a)	Healthcare	AI (unspecified)	C, E, T
(Sonntag et al., 2023)	Consumer	Other (Virtual Reality)	C, E, T
(Thornton et al., 2019)	Transportation	Autonomous Systems Algorithm	C, E, T
(Tsunetomo et al., 2022)	Consumer	Other (Robotics)	C, E, T
(Tuomela et al., 2019)	Consumer	Other (Smart Home)	E
(Umbrello et al., 2024)	Computation	Other (Quantum Technologies)	C
(Umbrello, 2020)	Consumer	AI (unspecified)	C, T
(van der Hoek S et al., 2023)	Industry	Other (Augmented Reality)	C, E, T
(Vernim et al., 2022)	Industry	AI (unspecified)	C, E, T
